# Single-cell transcriptome dataset of human and mouse in vitro adipogenesis models

**DOI:** 10.1038/s41597-023-02293-x

**Published:** 2023-06-16

**Authors:** Jiehan Li, Christopher Jin, Stefan Gustafsson, Abhiram Rao, Martin Wabitsch, Chong Y. Park, Thomas Quertermous, Joshua W. Knowles, Ewa Bielczyk-Maczynska

**Affiliations:** 1 Division of Cardiovascular Medicine, Department of Medicine, Stanford University School of Medicine, Stanford, CA 94305 USA; 2 Stanford Diabetes Research Center, Stanford University School of Medicine, Stanford, CA 94305 USA; 3 Stanford Cardiovascular Institute, Stanford University School of Medicine, Stanford, CA 94305 USA; 4 Clinical Epidemiology Unit, Department of Medical Sciences, Uppsala University, Uppsala, Sweden; 5 Department of Bioengineering, Stanford University, Stanford, CA 94305 USA; 6 Department of Pediatrics and Adolescent Medicine, Center for Rare Endocrine Diseases, Division of Pediatric Endocrinology and Diabetes, Ulm University Medical Centre, Ulm, 89075 Germany; 7 Stanford Prevention Research Center, Stanford University School of Medicine, Stanford, CA 94305 USA

**Keywords:** Transcriptomics, High-throughput screening, Differentiation

## Abstract

Adipogenesis is a process in which fat-specific progenitor cells (preadipocytes) differentiate into adipocytes that carry out the key metabolic functions of the adipose tissue, including glucose uptake, energy storage, and adipokine secretion. Several cell lines are routinely used to study the molecular regulation of adipogenesis, in particular the immortalized mouse 3T3-L1 line and the primary human Simpson-Golabi-Behmel syndrome (SGBS) line. However, the cell-to-cell variability of transcriptional changes prior to and during adipogenesis in these models is not well understood. Here, we present a single-cell RNA-Sequencing (scRNA-Seq) dataset collected before and during adipogenic differentiation of 3T3-L1 and SGBS cells. To minimize the effects of experimental variation, we mixed 3T3-L1 and SGBS cells and used computational analysis to demultiplex transcriptomes of mouse and human cells. In both models, adipogenesis results in the appearance of three cell clusters, corresponding to preadipocytes, early and mature adipocytes. These data provide a groundwork for comparative studies on these widely used *in vitro* models of human and mouse adipogenesis, and on cell-to-cell variability during this process.

## Background & Summary

Adipose tissue carries out multiple roles that affect whole-body metabolism. In addition to storing energy in the form of lipids, it contributes to the homeostatic maintenance of blood glucose levels by taking up glucose in response to insulin and regulates the function of other metabolic organs by secreting hormones such as leptin and adiponectin^1,2^.

Adipogenesis is a differentiation process in which fat-specific progenitor cells (preadipocytes) convert into adipocytes, which carry out key metabolic functions of the adipose tissue. *In vivo*, preadipocytes are located in proximity of blood vessels within adipose tissue and contribute to adipose tissue maintenance and expansion in obesity^3^. Dysregulation of adipogenesis can result in metabolic disease, including insulin resistance and type 2 diabetes^4^.

Several preadipocyte *in vitro* models are routinely used to study the molecular regulation of adipogenesis. The most commonly used *in vitro* models include the immortalized mouse 3T3-L1 cell line^5^ and the primary, non-immortalized, non-transformed human Simpson-Golabi Behmel syndrome (SGBS) cell line^6^. These cellular models brought on major breakthroughs in our understanding of molecular mechanisms of adipogenic differentiation, both in development and in obesity^7,8^. However, adipogenic models show high levels of cell-to-cell heterogeneity in their differentiation responses to stimuli^9^. This heterogeneity can be due to multiple factors, including variations in preadipocyte commitment and stochasticity of responses to differentiation stimuli. Despite that, adipogenesis is often studied using bulk approaches, such as bulk RNA-Sequencing, which ignore the variability between individual cells, likely masking the presence of distinct cell subpopulations during adipogenesis.

**Fig. 1:**
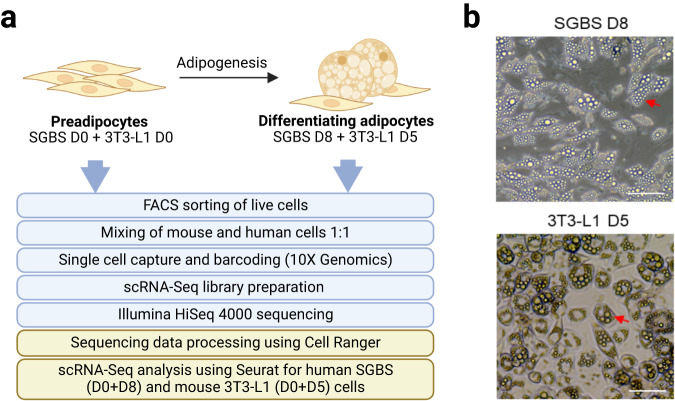
scRNA-Seq of mouse and human adipogenesis. (**a**) Schematic of the workflow. Human SGBS and mouse 3T3-L1 cells were analyzed at two time points, corresponding to before (D0) and during (D5 for 3T3-L1, D8 for SGBS) adipogenesis. At each time point, live cells were purified using exclusion of propidium iodide-stained cells by FACS. Equal numbers of SGBS and 3T3-L1 cells were then mixed and subjected to microfluidic single-cell capture with GelBeads-in-emulsion (GEMs) using 10X Chromium Controller. Single-cell cDNA libraries were prepared using the Chromium Single Cell 3′ Library & Gel Bead Kit (10X Genomics), followed by sequencing on Illumina HiSeq 4000. Computational analysis involved barcode processing, UMI counting, demultiplexing, gene and cell filtering, normalization, and clustering. (**b**) Representative light microscopy images of differentiated cells, SGBS at day 8 (D8) and 3T3-L1 at day 5 (D5), show similar pattern of lipid deposition in adipocytes (arrows). Scale bar: 50 µm.

Here, we present a single-cell RNA-Sequencing (scRNA-Seq) dataset collected before and during adipogenic differentiation of 3T3-L1 and SGBS cells to allow for analyses of heterogeneity of transcriptional states before and during adipogenesis, as well as comparisons between mouse and human models of adipogenesis. To minimize technical variation, at two time points (before and during adipogenic differentiation) mouse and human cells were mixed in equal ratios and subjected to scRNA-Seq, followed by computational demultiplexing and separation of data from mouse and human cells (Fig. 1a). The time points were selected based on previously established time course comparison of adipogenesis in SGBS and 3T3-L1 cells^10^ and validated using light microscopy (Fig. 1b). Analysis of cells at later timepoints was not feasible due to the fragility of large adipocytes, which would preclude single-cell analysis. Through technical validation, we demonstrate quality of this dataset. By unsupervised clustering we identify cell populations that correspond to preadipocytes, differentiating and mature adipocytes in both models.

This dataset complements recent advances in characterizing the transcriptome of adipose tissue in human and mice at a single-cell^11–14^ and single-nucleus level^15^, which revealed significant level of transcriptional heterogeneity within both adipose progenitor cells and adipocytes. In addition, the progress in adipocyte cell culture led to establishment of new models with improved translational relevance over cell lines. For example, our dataset can be used as a point of reference for the investigation using other models of adipogenesis, including primary adipocyte precursor cells (APCs)^16,17^ and adipose mesenchymal stem cells (AMSCs)^18,19^. In addition, transcriptome data from differentiated SGBS and 3T3-L1 cells can be utilized to compare with *in vitro* adipocyte biology models, such as cultured primary adipocytes^20^.

## Methods

### Cell culture

The 3T3-L1 preadipocyte cell line was maintained in Dulbecco’s Modified Eagle’s Medium (DMEM, Thermo Fisher) with 10% Fetal Bovine Serum (GeminiBio, lot #A22G00J), 100 units/ml penicillin and 100 µg/ml streptomycin, in a humidified 5% CO2 incubator. Cells were used at passage 7. For adipogenic differentiation cells were grown to confluency. 48 h past confluency, some of the cells were collected for scRNA-Seq analysis before adipogenesis (day 0, D0), while other cells were differentiated by stimulation with 1 µM dexamethasone, 0.5 mM IBMX, 10 µg/ml insulin in growth medium. After 48 h the medium was changed to growth medium with 10 µg/ml insulin in growth medium until day 5 (D5), when the cells were collected for scRNA-Seq analysis during adipogenesis.

The SGBS cell line was cultured and differentiated as previously described^6^, and used at passage 34. Cells were maintained in a humidified chamber at 37 °C with 5% CO_2_, and the media was replaced every 2-3 days. The standard culture media used was composed of DMEM/Nutrient Mix F-12 (Invitrogen), supplemented with 33 uM biotin, 17 uM pantothenic acid, 10% FBS and antibiotics (100 IU/ml penicillin and 100 ug/ml streptomycin). Cells were cultured for three days post-confluence, and either subjected to scRNA-Seq (D0, before differentiation) or differentiated. Differentiation was induced by the change of culture media to DMEM/F-12, 33 uM biotin, 17 uM pantothenic acid, 0.01 mg/ml human transferrin, 100 nM cortisol, 200 pM triiodothyronine, 20 nM human insulin (Sigma-Aldrich), 25 nM dexamethasone, 250 uM IBMX, 2 uM rosiglitazone, and antibiotics. After four days of differentiation, the medium was replaced with DMEM/F-12, 33 uM biotin, 17 uM pantothenic acid, 0.01 mg/ml human transferrin, 100 nM cortisol, 200 pM triiodothyronine, 20 nM human insulin and antibiotics. SGBS cells were cultured for eight days after the induction of differentiation and subjected to scRNA-Seq analysis (time point during differentiation, D8).

Both cell types were cultured in 6-well polystyrene tissue culture plates (Falcon, #353046).

### Microscopy

Cultured cells were imaged using EVOS XL Core (Thermo Fisher Scientific) at 20X magnification.

### Single-cell sorting and cDNA library preparation

On the day of collection, cells were detached from culture plates using TrypLE Select Enzyme (Gibco), centrifuged at 300 × g for 5 min and resuspended in PBS with 0.04% Bovine Serum Albumin. Lack of staining with Trypan Blue Solution (Gibco) was used to sort live cells using Influx sorter (Beckman Dickinson), with >95% of single cells quantified as live in all experiments. Equal numbers of sorted live SGBS and 3T3-L1 cells were mixed and subjected to single-cell capture on the 10X Chromium Controller device at Stanford Genomics Service Center during which single cells were encapsulated with individual Gel Beads-in-emulsion (GEMs) using the Chromium Single Cell 3′ Library & Gel Bead Kit (10X Genomics). The number of cells targeted in each experiment was 10,000, following manufacturer’s guidelines. In-drop reverse transcription and cDNA amplification was conducted according to the manufacturer’s protocol to construct expression libraries. Library size was checked using Agilent Bioanalyzer 2100 at the Stanford Genomics facility. The libraries were sequenced using Illumina HiSeq 4000.

### Raw data processing

Cell Ranger v2.10 was used for processing and analysing the raw single cell FASTQ files. The following genome builds were used: mm10 for the mouse genome, hg19 for the human genome. Quality control (QC) steps that were taken to assess the quality of the sequencing data and to identify potential included: sample demultiplexing, read alignment and filtering, gene expression quantification, cell filtering and QC metrics, and data normalization and batch correction. The batch correction was performed with the Seurat base function “MergeSeurat”. 10,198 cells passed the QC when D0 SGBS and D0 3T3-L1 cells were analysed, compared to 6,785 cells when D5 3T3-L1 and D8 SGBS cells were analysed. Only reads mapping to mm10 or hg19 were used for downstream processing. Genome mapping was used to assign each cell as either human or mouse.

### Bioinformatic analysis of scRNA-Seq data

Seurat v4.3^21^ was used to merge processed data for two single-cell sequencing runs, combining sequencing data from different stages of adipocyte differentiation. The data was first split between human and mouse data, pre-processed using Seurat, then log normalized. The major variable features within the processed data were identified using Variance Stabilizing Transformation. The gene matrix was then visualized and analysed using principal component analysis (PCA), with gene associations to each principal component displayed. Seurat’s *FindNeighbors* and *FindClusters* functions (resolution = 0.09) were used to identify groups within the samples. The data were further visualized via the PCA, Uniform Manifold Approximation and Projection (UMAP), and t-distributed Stochastic Neighbor Embedding (t-SNE) dimensional reduction techniques. Seurat’s *FindAllMarkers* function identified genes specific to each cluster, with previous annotations indicating that genes were clustered by stages in cell differentiation. Feature plots for specific differentiation features were visualized in a t-SNE plot and through heatmaps for each cluster using Seurat’s *DoHeatMap* and *FeaturePlot* functions. Pseudotime analysis was performed using the Slingshot package in R to visualize the cell differentiation process. To visualize the overlap in cell markers between human and mouse cells, the Euler package was used to generate a Venn diagram.

## Data Records

Sequencing data have been submitted to the NCBI Gene Expression Omnibus (GSE226365)^22^. The dataset consists of raw sequencing data in FASTQ format, separated by the time point: D0 3T3-L1 and D0 SGBS (GSM7073976) and D5 3T3-L1 and D8 SGBS (GSM7073977). In addition, we provide processed data, separated by time point and cell line, including *barcodes.tsv*, *genes.tsv* and *matrix.mtx* files, listing raw UMI counts for each gene (feature) in each cell (barcode) in a sparse matrix format as supplementary files. R Data files for processed Seurat data objects, gene marker tables, and quality control summaries can be found in the GEO submission^22^ and on the github repository.

## Technical Validation

To validate the quality of our data, we investigated the technical quality and the unsupervised clustering and its reproducibility between the two datasets.

### Quality control of the scRNA-Seq dataset

**Fig. 2:**
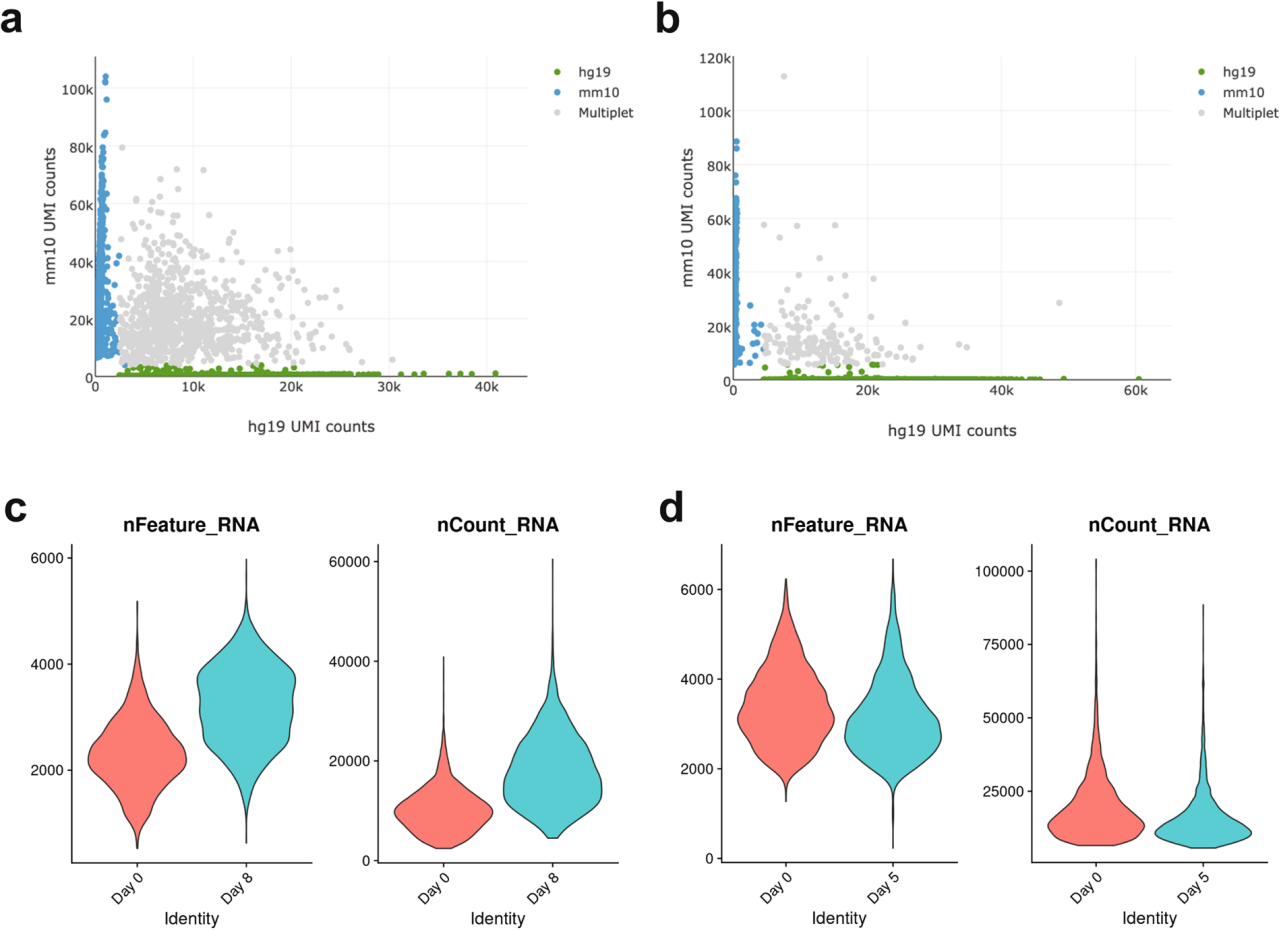
Single-cell RNA-Seq dataset quality assessment following inferring the species from transcriptome. (**a,b**) Plots representing quantification of the alignment of individual cell’s transcriptomes to the human (hg19) and mouse (mm10) genomes at (**a**) D0 3T3-L1/D0 SGBS, and (**b**) D5 3T3-L1/D8 SGBS. (**c,d**) Violin plots of gene counts (nFeature_RNA) and UMI counts (nCount_RNA) after quality control filtering in (**c**) SGBS cells and (**d**) 3T3-L1 cells, separated by the day of differentiation.

Interpretation of single-cell transcriptomics data is highly sensitive to technical artifacts. Sequencing data alignment using Cell Ranger led to the identification of comparable numbers of human and mouse cells within each of the analysed time points, as expected (Fig. 2a,b, Table 1). We used further steps to filter cells, removing any multiplets and cells with fewer than 200 genes detected (Fig. 2c,d, Table 2).

**Table 1:** Detailed QC report of 10X Genomics sequencing files (Cell Ranger).

Raw sequencing sample	SGBS D0, 3T3-L1 D0		SGBS D8, 3T3-L1 D5	
Number of reads	320,829,287		334,091,518	
Q30 bases in barcodes	96.9%		97.5%	
Q30 bases in RNA reads	76.7%		77.4%	
Q30 bases in UMI reads	96.8%		97.6%	
Mean reads per cell	31,460		49,239	
**Processed sample**	**SGBS D0**	**3T3-L1 D0**	**SGBS D8**	**3T3-L1 D5**
Reads mapped to genome	30.8%	58.1%	51.1%	41.7%
Reads mapped to exons	25.6%	46.8%	43.2%	33.7%
Reads mapped uniquely to genome	29.8%	52.6%	49.8%	38.5%
Estimated number of cells	5,672	5,402	3,655	3,305
Fraction of reads in cells	94.40%	94.50%	93.2%	93.3%
Median genes per cell	2,239	3,360	3,199	3,011
Total genes detected	19,339	17,013	19,862	16,444

**Table 2:** Final cell quantification statistics.

	SGBS D0	SGBS D8	3T3-L1 D0	3T3-L1 D5
Unfiltered cells	5,672	3,655	5,402	3,305
Filtered cells	4,742	3,480	4,526	3,118
Filtered genes detected	16,486	17,178	14,755	14,436

### Annotation of cell subpopulations

**Fig. 3:**
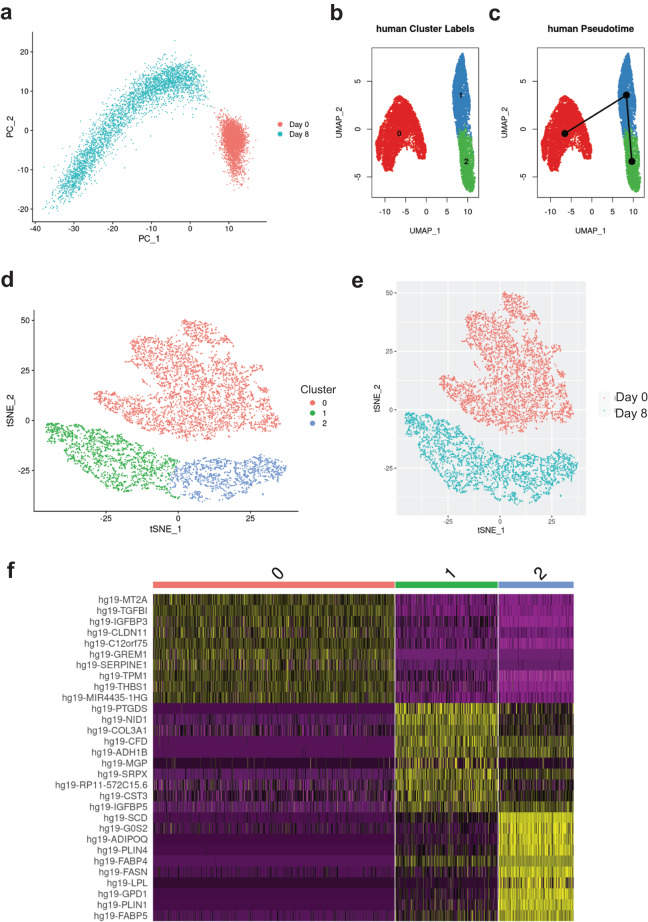
Clustering of scRNA-Seq data in human SGBS cells. (**a**) Primary component analysis (PCA) plot. (**b**) UMAP plot. (**c**) Pseudotime analysis. (**d**) t-SNE plot. (**e**) Assignment of cells by differentiation day (D0 vs. D8), superimposed on the t-SNE plot. (**f**) Heatmap showing the expression of top 10 enriched genes per cell cluster.

**Fig. 4:**
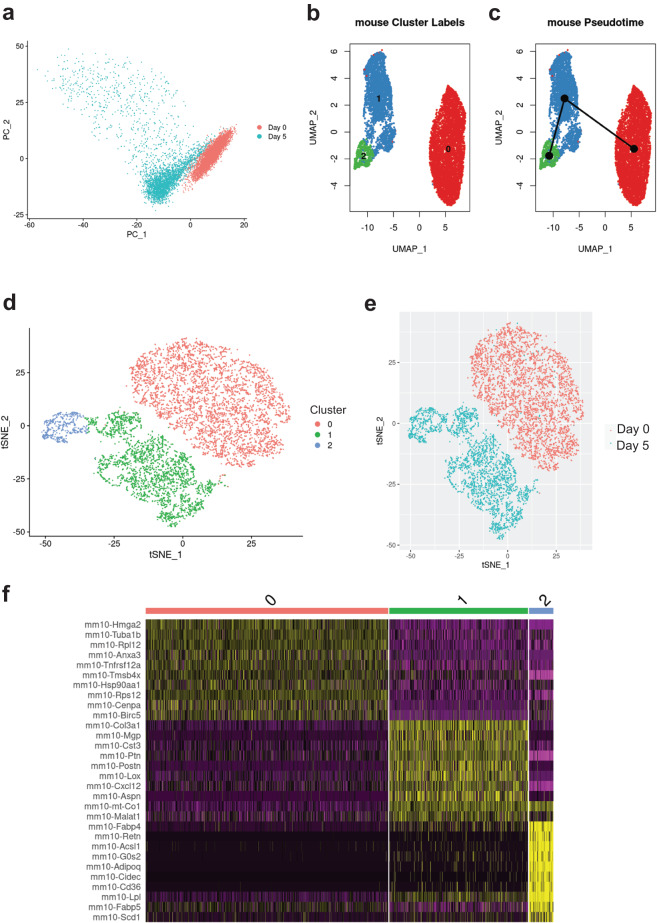
Clustering of scRNA-Seq data in murine 3T3-L1 cells. (**a**) Primary component analysis (PCA) plot. (**b**) UMAP plot. (**c**) Pseudotime analysis. (**d**) t-SNE plot. (**e**) Assignment of cells by differentiation day (D0 vs. D5), superimposed on the t-SNE plot. (**f**) Heatmap showing the expression of top 10 enriched genes per cell cluster.

**Fig. 5:**
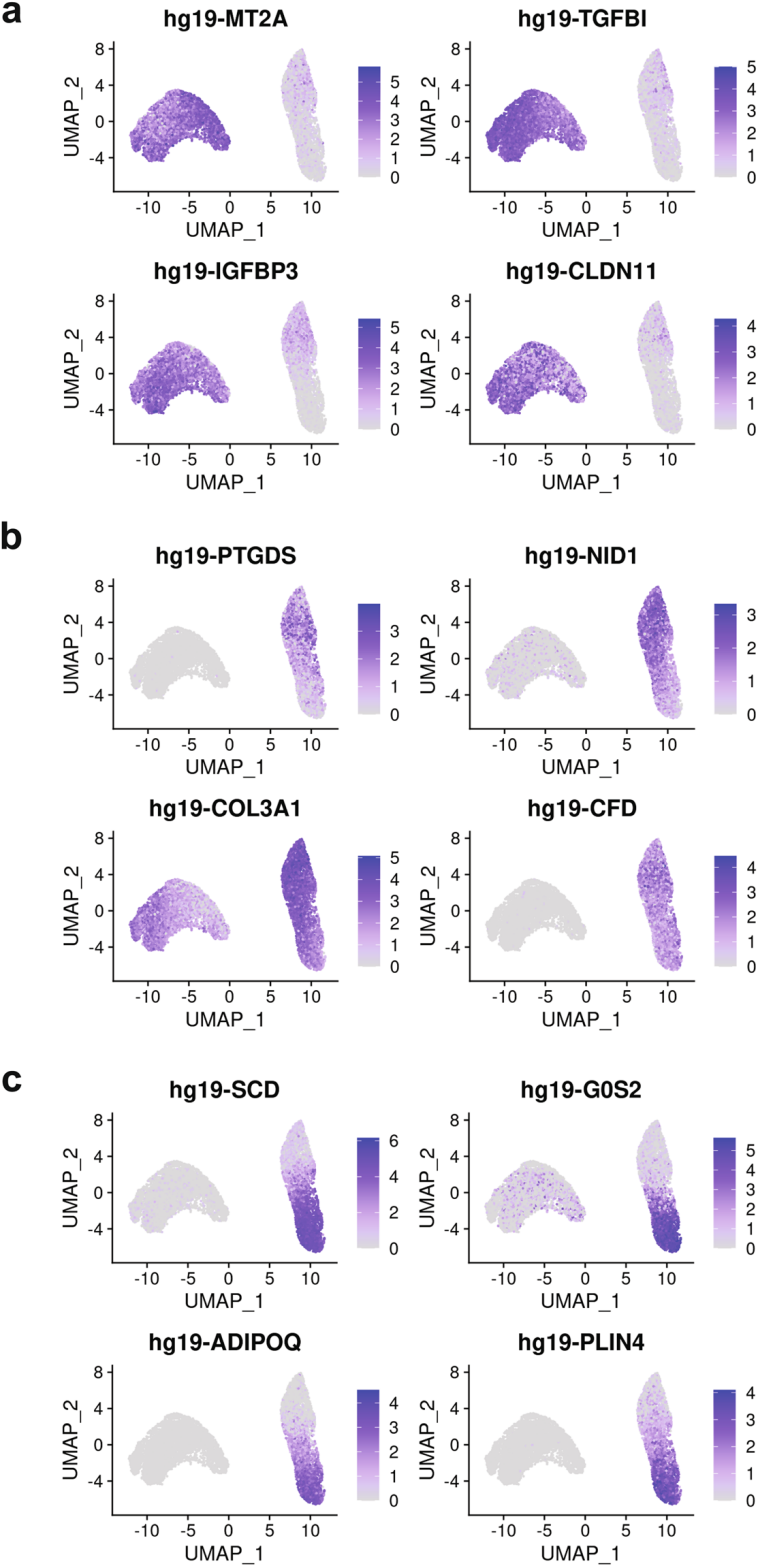
Feature plots showing the expression of cluster marker genes for individual clusters in human SGBS cells. (**a**) Cluster 0 (preadipocytes); (**b**) Cluster 1 (differentiating); (**c**) Cluster 2 (adipocytes).

**Fig. 6:**
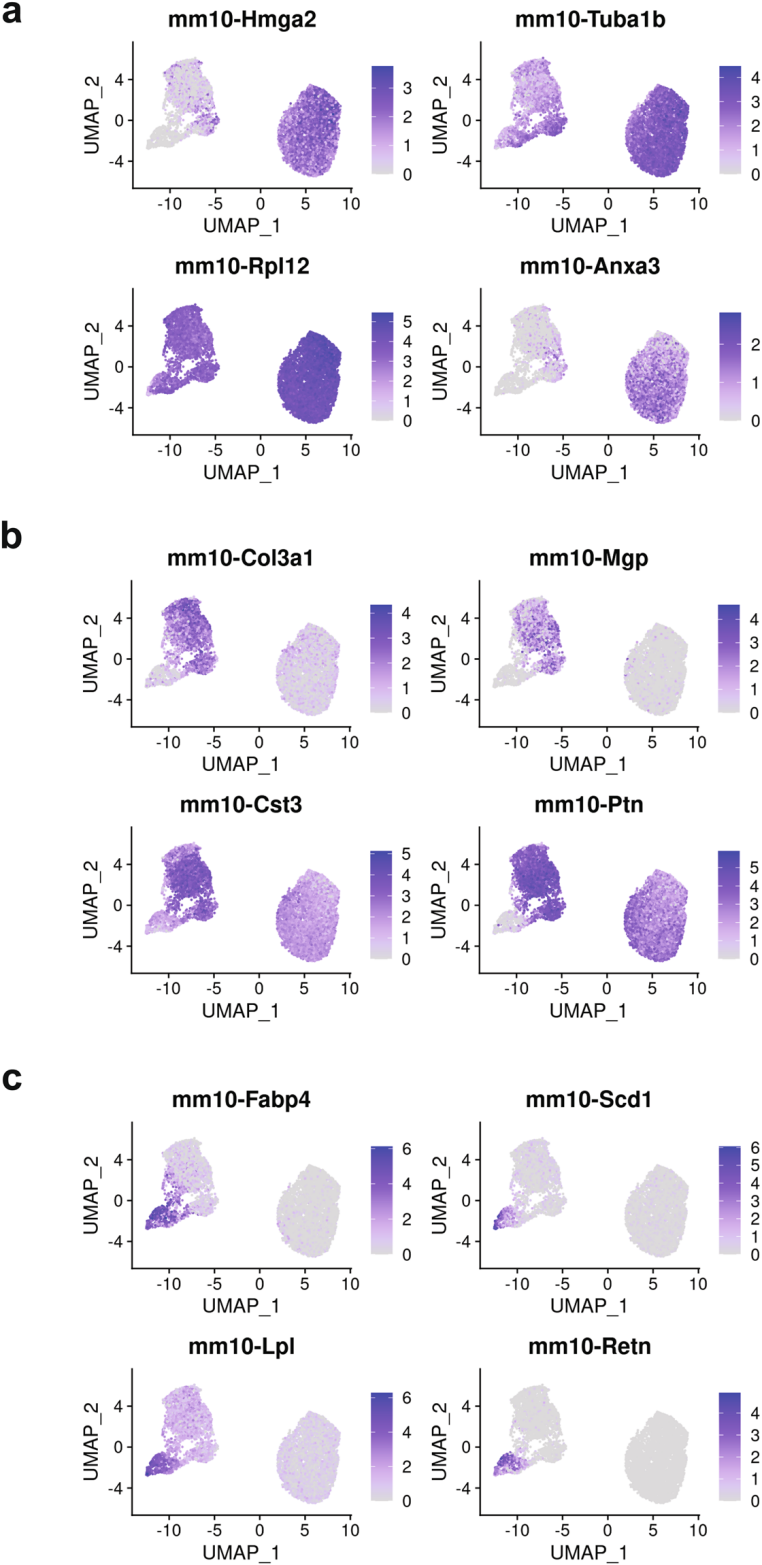
Feature plots showing the expression of cluster marker genes for individual clusters in mouse 3T3-L1 cells. (**a**) Cluster 0 (preadipocytes); (**b**) Cluster 1 (differentiating); (**c**) Cluster 2 (adipocytes).

**Fig. 7:**
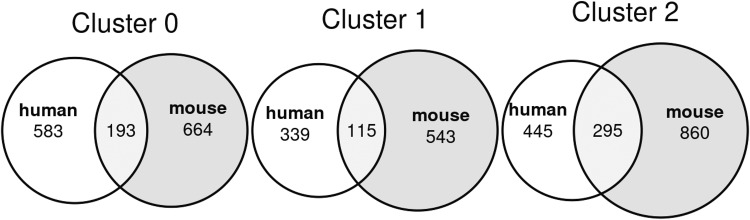
Venn diagram representation of the number of unique and shared marker genes between SGBS and 3T3-L1 cell lines, separated by cell cluster.

Adipogenesis is a highly heterogeneous process, and we expected the addition of differentiation stimuli to result in the appearance of additional cell states compared to D0 of differentiation, prior to the exposure to differentiation media. In fact, for both 3T3-L1 and SGBS cells we identified three cell clusters whose transcriptional profiles suggest they are preadipocytes, differentiating cells and adipocytes, which is supported by the pseudotime analysis (Figs. 3a–c, 4a–c). Furthermore, in both cell models there was a clear separation of cells isolated at D0, which corresponded to the preadipocyte clusters, and cells isolated after the induction of adipogenesis (D5 in 3T3-L1, D8 in SGBS), which corresponded to the other clusters (Figs. 3d,e, 4d,e). Our scRNA-Seq dataset includes cells collected at two separate timepoints and processed independently, therefore we cannot rule out the presence of a batch effect contributing to the separation of D0 cells from later time points, which is a limitation of this study. However, analysis of the genes enriched in the identified cell clusters supports the view that the treatment with differentiation media affects the transcriptome, regardless of whether the cells fully differentiate, resulting in the differences between the clusters at D0 and D5/D8. In particular, adipogenesis is associated with major changes in the composition of the extracellular matrix (ECM) components. In line with previously published work, the preadipocyte cluster in SGBS cells showed enrichment in the expression of claudin 11 (*CLDN11*)^23^, and the clusters containing differentiating cells both in SGBS and 3T3-L1 models showed an enrichment of the expression of collagen type III alpha 1 chain (*COL3A1*, *Col3a1*) which is associated with adipogenic differentiation^24^. Furter, adipocyte markers fatty acid binding protein 4 (*FABP4*)^25,26^, adiponectin (*ADIPOQ*)^27^, and perilipin 4 (*PLIN4*)^28^ were identified in the SGBS adipocyte cluster and *Fabp4*^25,26^, lipoprotein lipase (*Lpl*)^29^, and resistin (*Retn*)^30^ were identified in the 3T3-L1 adipocyte cluster (Figs. 3f, 4f, 5–7, Table 3). Full list of marker genes is provided as a.csv file with the GEO submission (#GSE226365)^22^.

**Table 3:** Description of cell clusters identified by unsupervised clustering.

Cell line	Cluster number and description	Top 5 enriched genes	Number of cells	% All cells
SGBS	0 – preadipocytes	*MT2A*, *TGFBI*, *IGFBP3*^31^, *CLDN11*^23^*, C12orf75*	4,744	57.70
	1 – differentiating	*PTGDS*^32^, *NID1*^33^, *COL3A1*^24^, *CFD*^34^, *ADH1B*^35^	2,002	24.35
	2 – adipocytes	*SCD*^36^, *G0S2*^37^, *ADIPOQ*^27^, *PLIN4*^28^, *FABP4*^25,26^	1,476	17.95
3T3-L1	0 – preadipocytes	*Hmga2*^38^, *Tuba1b*, *Rpl12*, *Anxa3*^39^, *Tnfrsf12a*^40^	4,574	59.84
	1 – differentiating	*Col3a1*^24^, *Mgp*^41^, *Cst3*, *Ptn, Postn*	2,612	34.17
	2 – adipocytes	*Fabp4*^25,26^, *Scd1*^36^, *Lpl*^29^, *Retn*^30^, *Acsl1*^42^	458	5.99

## Data Availability

All analytical code used for processing and technical validation is available on the GitHub Repository (https://github.com/christopherjin/SGBS_3T3-L1_differentiation_scRNASeq). The provided R code was run and tested using R 4.2.2.
